# The Need for Improved Detection and Management of Adult-Onset Hearing Loss in Australia

**DOI:** 10.1155/2013/308509

**Published:** 2013-04-28

**Authors:** Catherine M. McMahon, Bamini Gopinath, Julie Schneider, Jennifer Reath, Louise Hickson, Stephen R. Leeder, Paul Mitchell, Robert Cowan

**Affiliations:** ^1^Centre for Language Sciences, Australian Hearing Hub, 16 University Dve, Macquarie University, North Ryde, NSW 2109, Australia; ^2^HEARing Cooperative Research Centre, 550 Swanston St, Audiology, Hearing and Speech Sciences University of Melbourne, VIC 3010, Australia; ^3^Centre for Vision Research, Department of Ophthalmology and Westmead Millennium Institute, The University of Sydney, Westmead Hospital, Westmead, NSW 2145, Australia; ^4^Menzies Centre for Health Policy, Victor Coppleson Building, The University of Sydney, NSW 2006, Australia; ^5^School of Medicine, University of Western Sydney, Penrith, NSW 2751, Australia; ^6^School of Health and Rehabilitation Sciences, St Lucia Campus, University of Queensland, Brisbane, QLD 4072, Australia; ^7^School of Audiology, 550 Swanston St, Audiology, Hearing and Speech Sciences, University of Melbourne, VIC 3010, Australia

## Abstract

Adult-onset hearing loss is insidious and typically diagnosed and managed several years after onset. Often, this is after the loss having led to multiple negative consequences including effects on employment, depressive symptoms, and increased risk of mortality. In contrast, the use of hearing aids is associated with reduced depression, longer life expectancy, and retention in the workplace. Despite this, several studies indicate high levels of unmet need for hearing health services in older adults and poor use of prescribed hearing aids, often leading to their abandonment. In Australia, the largest component of financial cost of hearing loss (excluding the loss of well-being) is due to lost workplace productivity. Nonetheless, the Australian public health system does not have an effective and sustainable hearing screening strategy to tackle the problem of poor detection of adult-onset hearing loss. Given the increasing prevalence and disease burden of hearing impairment in adults, two key areas are not adequately met in the Australian healthcare system: (1) early identification of persons with chronic hearing impairment; (2) appropriate and targeted referral of these patients to hearing health service providers. This paper reviews the current literature, including population-based data from the Blue Mountains Hearing Study, and suggests different models for early detection of adult-onset hearing loss.

## 1. Introduction

Adult-onset hearing loss is a highly prevalent yet relatively underrecognised health problem in the older adult Australian population [1, 2]. Because hearing loss is often progressive and gradual in its onset in most individuals, it is typically diagnosed and managed several years after its onset, often only after having led to multiple negative consequences including effects on employment, poor quality of life, social isolation, depressive symptoms, increased mortality risk, and reduced independence [3–9]. It is one of the leading causes of burden of disease prior to older age, for ages 45–64 years, in men and women [9]. Further, as hearing loss interferes with so many of life's activities, it may prove to be a major impediment to society's need to have people remain longer in the workforce as the proportion of “working age” people in developed countries shrinks [10]. In Australia, the annual cost of lost earnings due to workplace separation and early retirement from hearing loss was estimated at $6.7 billion, which is over half of the calculated economic impact of hearing loss ($11.75 billion, representing 1.4% of GDP) [11]. Therefore there is a need to better understand the barriers that may exist to help seek an effective remediation for hearing loss in this population.

Hearing loss is a chronic problem and, contrary to current community perception and funding models of hearing services, hearing aids are typically a part of a rehabilitation program rather than provide a single and simple restorative solution to hearing loss [12]. As such, hearing loss needs to be effectively managed under a biopsychosocial model of care [13], following the framework for intervention and treatment of the International Classification of Functioning Disability, and Health model [14]. This framework not only considers the impairment *per se*, but also the impact that it has on the individual in terms of activity limitations (such as inability to perceive speech in noisy environments) and participation restrictions (such as the ability to fully participate in communication and conversational activities) [15]. Nonetheless, hearing aid use is a measurable quantity and, therefore, the majority of studies that have evaluated functional and quality-of-life outcomes of rehabilitation programs for individuals with hearing loss have used this as a marker. Multiple studies have identified that rehabilitation interventions can effectively address many of the difficulties associated with impaired hearing [16–20]. Importantly, evidence shows that the later hearing rehabilitation occurs in the course of hearing loss, the less likely older adults are to continue to use and derive benefit from hearing aids [21]. Despite this, several studies [22, 23] indicate high levels of unmet need for hearing health services and poor use of prescribed hearing aids. “Denial” or nonacceptance of hearing loss and the stigma associated with hearing loss are factors associated with this reluctance to seek help. Other reasons include an underestimation of the negative impacts of hearing impairment on overall health by general practitioners (GPs) and older adults, leading to poor referral to appropriate medical and allied health practitioners, such as ear, nose, and throat specialists and audiologists [24]. 

To date, the Australian public health system does not have an effective and sustainable hearing loss screening strategy for late-onset hearing loss in adults to manage this problem. This paper aims to review the current pathway of detection, referral, and management of late-onset adult hearing loss in Australia and to identify an alternative, more effective pathway for the future. 

## 2. Prevalence, Incidence, and Risk Factors of Adult-Onset Hearing Loss in Australia

Australian population-based data describing prevalence, incidence, and risk factors for hearing loss have been identified in the Blue Mountains Hearing Study (BMHS) in 1997–2000 among 2956 participants of the Blue Mountains Eye Study (BMES) cohort (an overall response rate of 75.5% for the cross-section) [25, 26]. Of these, 870 participants without hearing loss and 439 with hearing loss were reexamined during 2002–2004. Hearing thresholds were measured in audiometric soundproof rooms by qualified audiologists and bilateral hearing loss was described by the pure-tone average of air-conduction thresholds at octave frequencies between 500 and 4000 Hz (PTA_0.5–4 kHz_) in the better ear. Any hearing loss was defined as PTA_0.5–4 kHz_ > 25 dBHL. Risk factors measured (either via self-report or practitioner measurement) included self-reported health, noise exposure, and family history of hearing loss. In this study, we identified that a 33.0% prevalence of bilateral hearing loss existed in persons aged 50+ years (51% showed hearing loss in the worse ear) consistent with that measured in the US-based Epidemiology of Hearing Loss Study (EHLS) [27]. More specifically, mild hearing loss was present in 22.4% of participants, moderate in 8.9% and severe in 1.7% participants. For each decade beyond age 50, prevalence of hearing loss doubled. Men were 40% more likely to have hearing loss than women. Further, a history of having worked in a noisy environment predicted a 70% increased likelihood of any hearing loss, whereas family history predicted a 68% increased risk of hearing loss, which increased with greater magnitudes of loss [28]. The overall 5-year progression of hearing loss, defined as a difference in PTA > 10 dB, was moderately high at 15.7%, with the highest rate being evident in adults aged 80 years or older [26]. Additionally, for each decade of age over 60 years, the risk of incident hearing loss increased threefold. 

As well as health-related influences, our epidemiological study also assessed quality-of-life and mental health factors, such as cognitive function and depression. BMHS-I data showed that bilateral hearing loss was associated with poorer SF-36 scores in both physical and mental domains (decrease in physical component score (PCS) of 1.4 points, *P* = 0.025; decrease in mental component score (MCS) of 1.0 point, *P* = 0.13); with poorer scores associated with more severe levels of impairment (PCS *P*_trend_ = 0.04, MCS *P*_trend_ = 0.003) [3]. BMHS participants with any hearing loss were 64% more likely to have depressive symptoms [4]. Persons with moderate-to-severe hearing loss had slightly lower mean cognitive function scores than those without hearing loss (*P* < 0.001) [29]. Therefore, while milder levels of hearing losses were significantly more common in working-aged older adults, a lack of responsiveness to manage this early can lead to significant negative effects on quality of life, personal relationships, and ability to continue to work effectively. As the risk of hearing loss increases with advancing age, it seems that early detection and management would be critical to minimising any longer-term effects.

## 3. Poor Recognition and Uptake of Hearing Services

**Figure 1:**
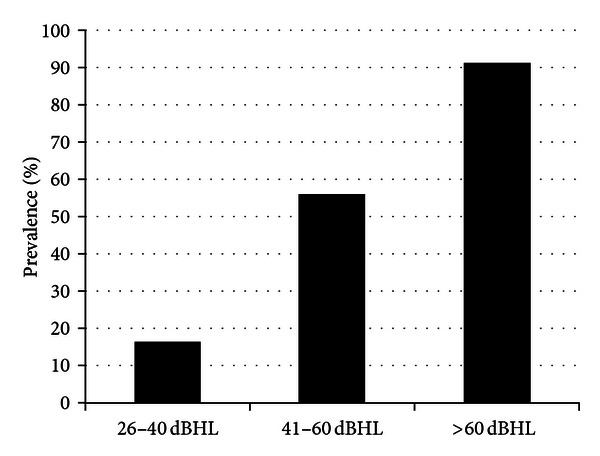
Prevalence of hearing aid ownership for individuals with a mild (26–45 dBHL), moderate (46–60 dBHL), and sever-profound (>60 dBHL) hearing loss. Data from Hartley et al. [35].

**Figure 2:**
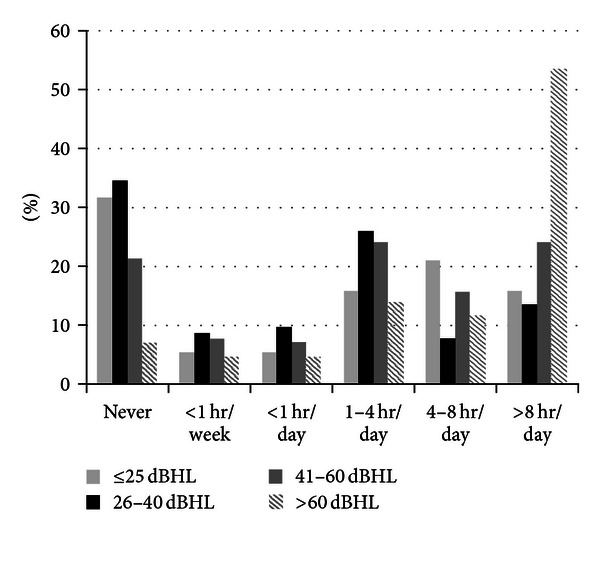
Percentage of time spent wearing hearing aids by magnitude of hearing loss in the better ear. Amended from Hartley et al. [35].

Stephens et al. [30] suggest that the average consumer presenting at a hearing aid or rehabilitation clinic for the first time is aged ~70 years and has had hearing problems for about 10 years. As hearing loss significantly impacts on communication ability [31] and communication is necessary for developing and maintaining effective relationships [32], it is likely that within this prolonged timeframe the individual and his/her family have experienced considerable frustration from the disability [33]. Hearing aids and associated rehabilitation programs have been shown to minimise such impacts. The US National Council on Aging survey of 2069 hearing-impaired individuals and 1710 of family and friends demonstrated that hearing aid use is associated with lesser degrees of anger and frustration reported by family members [12]. Further, Stark and Hickson [34] demonstrated benefits in hearing-related quality-of-life scales for both the individual with hearing loss and their significant other after hearing aid fitting, despite only 1/3 of the individuals with hearing loss showing initial motivation to attend the hearing appointment. Certainly, we found that BMHS participants who used their hearing aid at least 1 hour/day or more were only one-third as likely to report depressive symptoms as infrequent users, multivariate adjusted OR 0.32 (95% CI 0.14–0.76) [4]. Despite this, BMHS findings [34] showed that of 33.0% persons with measured bilateral hearing loss, only 33% owned hearing aids and, of these, only 25% used them habitually [3], similar to the rates of use reported in the EHLS study [27]. When stratified into magnitudes of hearing loss, BMHS data showed that hearing aids were owned by only 16.4% of individuals with a mild loss, compared with 55.8% with a moderate loss and 91.3% with a severe-profound loss (Figure 1) [35], suggesting that either there is a critical unmet need for hearing services in individuals with mild-moderate levels of hearing loss or that hearing aids are not needed for all individuals with lower magnitudes of loss or that the technology is too difficult to manage in this population. Nonetheless, BMHS data showed that 33.4% of older adults with average hearing levels greater than 40 dBHL in the better ear did not own a hearing aid [35]. While milder forms of hearing loss may be less correlated with hearing disability, Dillon [10] showed that more significant losses do show higher levels of benefit. Further, BMHS data demonstrate that 53.5% of older adults with severe losses wear their hearing aids for over 8 hours per day compared to 24% of those with moderate losses and 13.5% with mild losses [35], suggesting an increased need for amplification for greater magnitudes of loss (Figure 2).

Low rates for use of hearing services and hearing aids highlight barriers including cost [36] and/or reluctance by many to accept their hearing loss (or those without self-perceived hearing disability) [37]. However, similar low rates of hearing service uptake and device use have been observed in the Australian Federal Government Office of Hearing Services program [10], where hearing services are largely provided free of charge to eligible older adults. Therefore, we assume that under use of fitted aids by older adults in Australia may suggest either poor targeting of individuals with hearing loss or fitting at too late a stage for derived benefit. Substantial delays in accessing hearing services may impact effective hearing aid use because advancing age is associated with poorer auditory and cognitive processing, physical dexterity, and learning abilities making it more challenging to perceive sounds in competing noise environments, position a hearing aid in the ear, and to learn how to use new technology [38–41]. Additionally, there is an increased likelihood of other health problems coexisting so that the management of hearing loss may be considered less of a priority and prove to be burdensome. As the consequences of the hearing loss are more significant, this may lead to poorer motivation to manage the impairment and/or its impacts.

## 4. GP Hearing Screening Strategies

There remains a large proportion of hearing-impaired adults who would benefit from hearing aids but who decide not to seek help [21]. Further, while BMHS showed that approximately one-third of people aged ≥50 years with measured bilateral hearing loss reported seeking help from their general practitioner (GP), a random cross-sectional survey of GP activity in Australia between 2003 and 2008 identified that only approximately 3/1000 consultations for older adults included hearing loss management [24]. Similar studies of GPs undertaken in UK identified that the chance of referral to hearing services for older adults who reported hearing loss was only about 50% [42]. Screening and intervention programmes have been recommended to improve this situation [21, 43]. Screening programs are not systematically implemented throughout the Australian population, their success at meeting the needs of the target population is not assured, and they have no automatic link to action if the need for action is detected [44]. Audiograms conducted by trained audiologists in soundproof booths, are currently used to diagnose hearing impairment and largely determine whether or not an individual is offered hearing rehabilitation. Audiometry is expensive and may not necessarily be accessible to those needing it. Particularly for late-onset hearing loss, it provides little information about effects of hearing loss on everyday functioning [45, 46]. It is important to note that while hearing impairment is extremely common in older adults, not all are significantly disturbed by this. The BMHS findings collectively show that severe hearing disability is strongly associated with measured hearing loss, poorer QOL, and probable depression. This suggests that identifying hearing-related activity limitations and participation restrictions could potentially be effective in identifying persons more likely to have suffered an important impact from their hearing impairment and, thus, would be most likely to benefit most from using hearing aids. Self-perception of a hearing disability (e.g., increasing social isolation) can often be an important reason to seek aural rehabilitation. In fact, Dillon [10] showed that the benefits reported by individuals with hearing aids appear to be only weakly correlated with hearing loss, particularly for mild-moderate losses. This may in part explain why at least 20% of individuals fitted with hearing aids do not wear them. On the other hand, benefits are actually more highly correlated with initial motivation and perceived listening difficulty [10, 47, 48]. Thus, greater engagement by GPs in hearing health could potentially be a cost-saving strategy, as GPs are ideally placed to better motivate and identify older people with hearing loss disability, that is, those likely to benefit the most from a hearing aid, thereby improving the targeting of hearing-impaired patients for rehabilitation.

There exists a need for a readily accessible screening test assessing hearing disability which could more accurately identify rehabilitation need, rather than just measurement of hearing loss. Validated, self-administered questionnaires about hearing disability have been shown to detect functional hearing impairment accurately and, so, have been recommended as potential screening tools [49–52]. These can also be administered quickly without specialised training [53]. In particular, it has been suggested that primary care services could cost-effectively be used to identify hearing disability using targeted questions, possibly alongside other screening interventions [21, 43]. Previous work through UK GP-based case finding, which targeted people in the 50–65-year age group, showed that effective hearing aid use can be at least tripled [30, 54]. One study assessed the patient's take-up of hearing disability screening and the subsequent take-up of hearing aids as an intervention for hearing disability. Substantial benefits were reported in hearing aid benefit outcome inventories and moderate benefits in health utilities index and quality-of-life scores from amplification for this target group [21]. Another UK study of 604 GP patients, aged 50–65 years [30], showed that the first posting of hearing disability questionnaires detected 78% of those prepared to accept hearing aids for the first time. The possession of hearing aids rose from 7% (at baseline) to 24% (after intervention), and 6 months later the hearing aids were being used regularly. The authors concluded that simple questionnaires are effective in detecting hearing disability in older adults and that this intervention was acceptable by many of those reporting significant hearing difficulties. 

Given the pivotal role of the GP in the early identification and management of chronic health problems, at least in Australia, the implementation of a GP-based hearing screening program for adults >50 years of age would be beneficial in addressing this problem. Further, with the inadequacies of the medical model in the treatment of chronic health conditions and the move towards a model of patient-centred care, GPs are effectively placed to assist with the minimisation of the stigma associated with hearing loss and enhancing patient self-motivation to manage this [55]. Current research identifies a critical role for GPs in both detection and appropriate referral of many other disorders/diseases such as obesity [56–58]. However, several such studies identified that the knowledge and attitudes of GPs can be a major barrier to effective intervention within this process [38]. Hence, underlying reasons for low rates of GP involvement in hearing health could include lack of awareness/understanding of (a) simple tools to identify hearing loss and associated disability; (b) risk factors for age-related hearing loss and ways to use this information to identify at-risk patients; (c) adverse impacts caused by hearing loss on the mental and physical well-being of older adults (i.e., disability); and (d) the benefits of aural rehabilitation. 

Given the increasing prevalence and disease burden of undetected hearing loss in older adults and the availability of effective interventions (e.g., hearing aids and/or assisted listening devices), there are 3 potential critical roles for the GP in hearing health: (1) early identification of patients with age-related hearing loss, as well as recognition of whether any negative consequences/disability has resulted; (2) assistance in reducing the stigma of hearing loss and motivating patients to seek further help; and (3) appropriate referral of these patients to hearing health providers. This could be achieved by sensitising GPs to recognise at-risk individuals and providing targeted questions to identify hearing loss disability. 

We have identified an important role of GPs in the process of targeting individuals with late-onset hearing loss and referral; however, the challenge that remains is how to effectively increase GPs knowledge and practice behaviour in this area. Possibly the most obvious method is through development of a continuing medical education (CME) program that targets the impacts of hearing loss and remediation and provides a reliable method of hearing screening in adults. The evidence for good outcomes of CMEs measured by factors including increased knowledge and skills as well as altered attitudes and practice behaviours is varied and possibly depends partly on the learners and learning context [59]. A review of the literature has identified that while the quality of evidence is not high, generally CME provides a strategy that increases knowledge and may elicit a change in practice behaviour [60]. However, in a meta-analysis of the CME literature, Forsetlund and colleagues [61] report that education meetings are likely to only have a moderate effect on professional practice and a smaller improvement on patient outcomes. Despite this, Cook et al. [59] demonstrated that while Internet-based programs have a significant effect on knowledge and behaviour compared with no-intervention, there is limited evidence to suggest that it is superior to other methods of delivery of learning materials. Therefore it is possible that both educational meetings and Internet-based programs will have only a moderate impact in enhancing referrals to hearing healthcare providers. 

## 5. Telephone/Internet Screening Programs

An alternative method of screening of hearing loss and disability which does not require GP involvement is telephone and/or Internet screening using digits in noise [62–64], which provides a quick, effective, and relatively inexpensive technique to detect hearing loss in adults [62, 63]. In addition, this presumably has a broader reach than GP screening because of the program's accessibility to individuals in rural and remote areas where worldwide shortages of healthcare professionals and services exist [65]. Further, it provides information about the individual's hearing to the significant proportion of individuals who were not intending to see a GP or hearing healthcare provider (as shown in [64]). Smits and colleagues [62, 64] developed the first telephone screening test which was introduced into The Netherlands in 2003 as the National Hearing Test. The screening test used 23 monosyllabic digit triplets presented by a female speaker, adaptively varying in level by 4 dB (to determine audibility) and then 2 dB (to seek threshold) and embedded in a 73 dBA speech noise, shaped to match the long-term average speech spectrum. They estimated the average signal-to-noise ratio (SNR) for speech reception threshold (SRT; 50% correctly identified) and characterised normal hearing using a criterion of −4.1 dB SNR, insufficient hearing between −4.1 and −1.4 dB SNR, and poor hearing >−1.4 dB SNR. In 38 participants with varying levels of hearing [62], this screening test showed excellent test-retest reliability (<1 dB error), sensitivity (0.91), and specificity (0.93) when compared to an equivalent speech-in-noise test conducted under headphones and took approximately 3 minutes to complete. A similar telephone screening test “Telscreen” using digit triplets embedded in spectrally shaped noise was developed and implemented in Australia in 2007 [63]. The noise was amplitude modulated by a 20 Hz sinusoid and had gaps in the frequency spectrum to increase the sensitivity of this test to identify sensorineural hearing losses (described in [63]). Significant correlations were found between Telscreen and the individual's four-frequency pure-tone average (*r* = 0.77, *P* < 0.001) and Telscreen and the presence of subjectively rated disability (*r* = 0.65, *P* < 0.001). 

Smits and colleagues [64] demonstrated that over 50% of those referred to medical or other professional hearing healthcare in The Netherlands were compliant in following this advice. On the other hand, Meyer and colleagues [63] showed that only 36% of the 193 individuals who failed the Telscreen in Australia went on to receive medical or other professional support. It is not clear whether such differences in health-seeking behaviour are explained by cultural, social, or economic factors.

## 6. Speech-in-Noise Tests

Another hearing screening program is the use of an automated face-to-face monosyllabic speech-in-noise test which aims to evaluate hearing disability in adults. The speech understanding in noise (SUN) test was developed by Paglialonga and colleagues [66, 67] and has been evaluated in multiple nonclinical sites with varying levels of ambient noise showing good sensitivity up to 65 dBA. The SUN test presents monosyllabic vowel-consonant-vowel sounds in a 3-alternative forced-choice paradigm. The response is provided through a touch screen, thereby avoiding tester scoring errors, and takes approximately 2 minutes to evaluate both ears. Good associations were found between pure-tone audiometry and referral on the SUN test [66] which indicates the benefit of this test as a screening test for adult hearing loss.

## 7. Conclusions

Given the ageing demographic and increasing average life span in Western countries, chronic hearing loss is projected to increase. A renewed focus on targeting the provision of hearing rehabilitation to people with self-perceived hearing disability, rather than those with only measured hearing loss, may lead to better long-term retention and use of aids. Therefore, over time the costs saved by provision of an effective and better-targeted health intervention enabling improved daily functioning among older adults will no doubt demonstrate this strategy and will provide “value for money.”
